# Fabrication, Characterization and Response Surface Method (RSM) Optimization for Tetracycline Photodegration by Bi_3.84_W_0.16_O_6.24_- graphene oxide (BWO-GO)

**DOI:** 10.1038/srep37466

**Published:** 2016-11-18

**Authors:** Chengjie Song, Xinying Li, Liping Wang, Weidong Shi

**Affiliations:** 1 School of Chemistry and Chemical Engineering, Jiangsu University, Zhenjiang, 212013, P. R. China; 2 School of Environmental and Safety Engineering, Changzhou University, Changzhou, 213164, P. R. China

## Abstract

RSM is a powerful tool for optimizing photocatalytic processes. The BWO-GO photocatalysts have been successfully synthesized via inorganic-salt-assisted hydrothermal method. XRD, TEM, FESEM, HRTEM and STEM are used to characterize BWO-GO heterojunction. UV-vis, PL, ESR and radical scavenger experiments are used to explore the photocatalysis mechanism. The photocatalysts are evaluated by TC photodegradation under visible light irradiation. And the main active species in TC photodegradation is ·O_2_^−^. Response surface methodology is used to optimize three key independent operating parameters, namely photocatalyst dosage (X_1_), percentages of GO (X_2_) and reaction time (X_3_), for TC photodegradation. The central composite design (CCD) is used to conduct experiments. The results showed that TC removal is significantly affected by the synergistic effect of linear term of X_1_ and X_3_. However, the quadratic terms of X_1_^2^ and X_3_^2^ had an antagonistic effect on T removal. The obtained RSM model (R^2^ = 0.9206) shows a satisfactory correlation between experimental and predicted values of TC removal. The optimized conditions is of 0.3 g photocatalyst dosage, 1.49 wt% GO loaded percentage and 90 min reaction time. Under this condition, theoretical prediction removal is 80.22% and the actual removal is 78.43%.

Tetracycline (TC) is one of the most used antibiotic chemicals, which used in human and veterinary medicine. Traditionally, it was not regarded as environmental pollutants; however, its occurrence in aquatic systems has attracted more and more attention as biological impacts and potential risks to the environment, as well as to human health, have been reported^1^,^2^,^3^,^4^. Recently, photocatalytic TC degradation has attracted more and more concentration, as the photocatalysis technology is economic, environmental and nontoxic and owns considerable application. Various photocatalysts have been developed and applied into TC degradation, such as TiO_2_^5^, V_2_O_5_^6^, NaNbO_3_^7^ and g-C_3_N_4_^8^.

Recently, much attention has been given to a series of visible light active bismuth-based photocatalysts. Many Bi^3+^- and Bi^5+^-containing compounds have been found to possess a narrow band gap and exhibit visible light photocatalytic activity because of the hybridized O2p and Bi6s^2^ valence bands^9^,^10^,^11^,^12^, such as Bi_2_MoO_6_^13^, BiOI^14^, Bi_2_WO_6_^15^, Bi_2_O_2_CO_3_^16^. However, all these Bi-based photocatalysts don’t show great photocatalytic performance. In the Bi-based photocatalysts, there is a special one-Bi_3.84_W_0.16_O_6.24_ (BWO). It owns nonintegral atomic ratio and its formation is attributed to the different solubility of WO_4_^2−^ and (Bi_2_O_2_)^2+^ in precursor suspensions with various pH^17^. Though BWO is a promising photocatalyst in environmental application and owns great optimized prospect. In our previous work, the pure BWO octahedral-like nanostructures could been obtained via a simple, quick and eco-friendly microwave-assisted synthetic method^18^. They exhibited the very highly photocatalytic activities for the tetracycline degradation under visible light irradiation. GO, potential as a reaction platform (large specific surface area and outstanding electronic properties), particularly for catalysis, has also been broadly described in numerous reports^19^,^20^,^21^. GO can be regarded as graphene functionalized by carboxylic acid, hydroxyl and epoxide groups^22^,^23^,^24^. And it is favorable for tunable optical, conductive and chemical properties^25^,^26^. Though, GO has been widely used in photocatalysis for contaminant removal, such as GO/TiO_2_^27^,^28^, GO/ZnO^29^, GO/Ag^30^, GO/N-TiO_2_^31^ and so on. To our best knowledge, there are no reports about Bi_3.84_W_0.16_O_6.24_-GO (BWO-GO) and it’s a good candidate for contaminant removal.

Nowadays, the efforts in photocatalysis are almost in changing material properties to improve the photocatalytic activity. And few efforts in optimizing the photocatalytic conditions to improve the photocatalytic activity. The RSM has been proved to be a powerful statistical technique for obtaining optimum conditions for advanced oxidation processes and evaluating the interactions of mutually influencing parameters with a limited number of experiments^32^,^33^,^34^. Compared to the classical approach for the same number of estimated parameters, RSM cannot only evaluate the interactions effects among tested operating variables but also reduce the number of experiments to be undertaken^35^,^36^,^37^. In this article, for the first time, a BWO-GO composite photocatalyst is synthesized and its photocatalytic activity is studied. In the photoactivity evaluation via the photocatalytic degradation of TC under visible-light irradiation. The parameters varied are the photocatalyst concentration, loaded percentages of GO and reaction time. Three factor central composite design (CCD) combined with RSM is applied to optimize the response as the photodegradation percentage of TC using BWO-GO (photocatalyst concentration, percentages of GO and reaction time is varied during the degradation).

## Results and Discussion

### Characterization of BWO-GO

**Figure 1:**
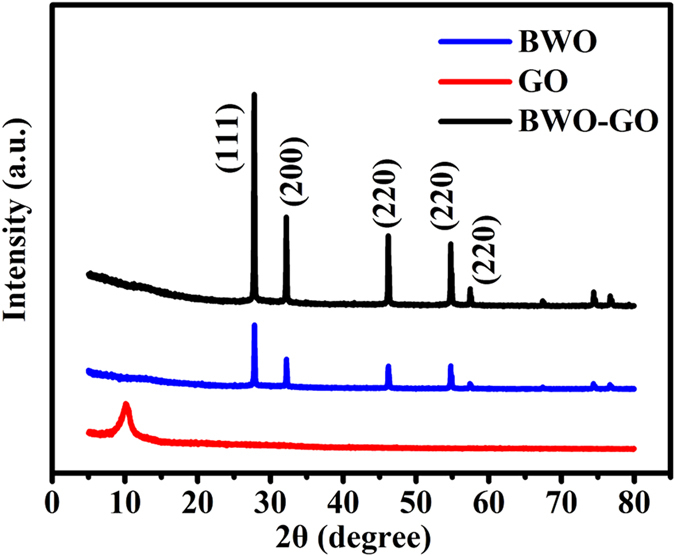
XRD patterns of GO, BWO and GO-BWO nanocomposites.

The XRD patterns of GO, BWO and BWO-GO nanocomposites are shown in Fig.1. The peaks in BWO could be readily indexed as JCPDS No. 00–043–0047. The lattice constants of BWO are a = 5.57 Å, b = 5.57 Å, and c = 5.56 Å. The characteristic peaks are sharp, implying the obtained nanostructures are well-crystallized. The XRD pattern of BWO-GO shows similar peak to pure BWO. Notably, no characteristics peaks of graphene or GO are observed in the composites. This is possibly due to two reasons: destruction of the regular stacking of GO due to microwave hydrothermal conditions^38^ and at relatively low graphene oxide loading in the nanocomposites, covering up of peaks of GO by the diffraction signals of BWO^39^.

**Figure 2:**
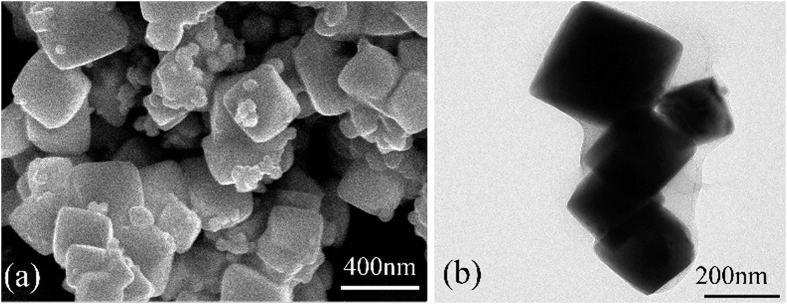
Images of the as-prepared BWO-GO. (**a**) FESEM; (**b**) TEM.

**Figure 3:**
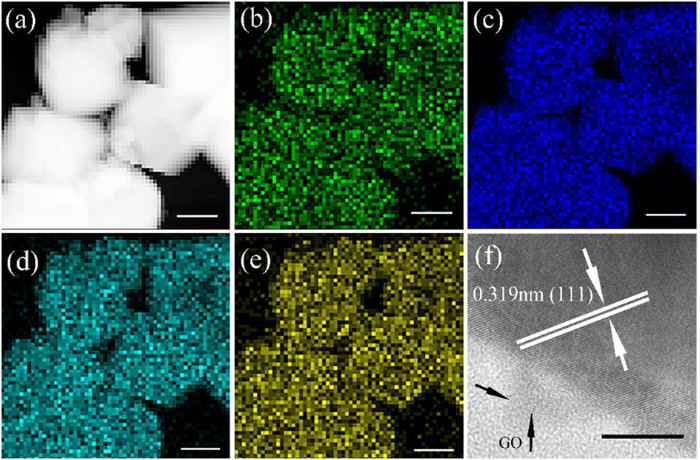
STEM image of BWO-GO. (**a**) STEM area, (**b**) W, (**c**) O, (**d**) Bi, (**e**) C. (**f**) HRTEM. Scale bar for (**a**), (**b**), (**c**), (**d**) and (**e**) is 100 nm, and for (**f**) is 10 nm.

The morphology and size of the products prepared by the procedure described in the experimental section are visualized by FESEM, HRTEM and STEM, as shown in Figs 2 and 3. As shown in low magnification (Fig. 2a), the FESEM results clearly indicate that the main component of the as-obtained BWO particle of about 200 nm, is uniform octahedral structure. As shown in TEM images (Fig. 2b), the BWO nanoparticles are deposited onto the graphene oxide sheets. This is due to the interaction between the hydrophilic functional groups of GO and BWO^40^.

To further investigate the micro-morphology and structure, HRTEM and STEM is employed. Figure 3 shows the STEM and HRTEM images of BWO-GO. In the mapping of W (Fig. 3b), O (Fig. 3c), Bi (Fig. 3d), C (Fig. 3e), it can be seen the homogeneous distribution of elements within the selected area. From analysis of high-resolution images as shown in Fig. 3f, the edge area of the octahedral nanoparticles exhibits well-resolved 2D lattice fringes. And it is possible to measure the lattice fringe spacing as being 3.19 Å, corresponding to the (111) planes of BWO.

**Figure 4:**
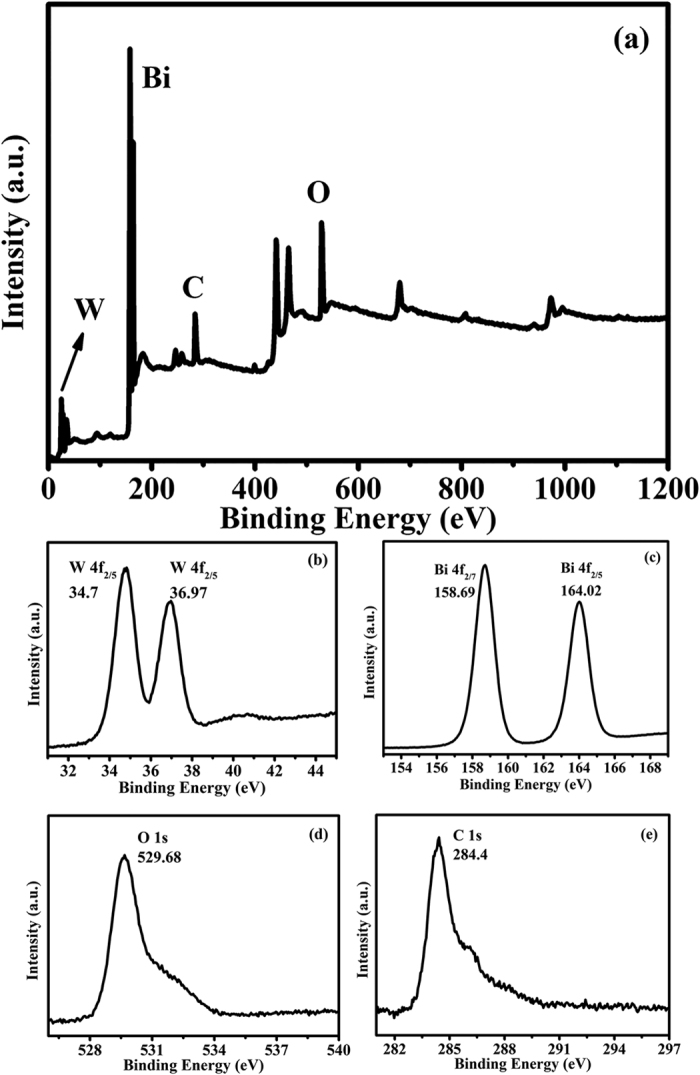
XPS scans of as-prepared 2 wt% BWO-GO. (**a**) full spectrum, (**b**) W4f, (**c**) Bi 4 f, (**d**) O 1 s and (**e**) C 1 s.

To check the elemental composition of the BWO-GO composite, X-ray photoelectron spectroscopy (XPS) was employed. Figure 4(a) shows the full XPS spectrum of BWO-GO, W, Bi, O and C elements. As shown in Fig. 4(b), the binding energies of 34.7 and 36.97 eV for W 4f_5/2_ peaks are attributed to W^6+^ in Bi_3.84_W_0.16_O_6.24_^41^. As displayed in Fig. 4(c), the characteristic binding energy values of 158.69 and 164.02 eV for Bi 4f_7/2_ and Bi 4f_5/2_ reveal a trivalent oxidation state for bismuth in Bi_3.84_W_0.16_O_6.24_^42^. The O 1 s binding energy 529.68 eV (crystal lattice oxygen) and C 1 s binding energies 284.4 eV in GO are also identified in the spectra (Fig. 4(d) and (e)). These results show evidence for the presence of GO in the ternary heterostructured BWO-GO composite.

**Figure 5:**
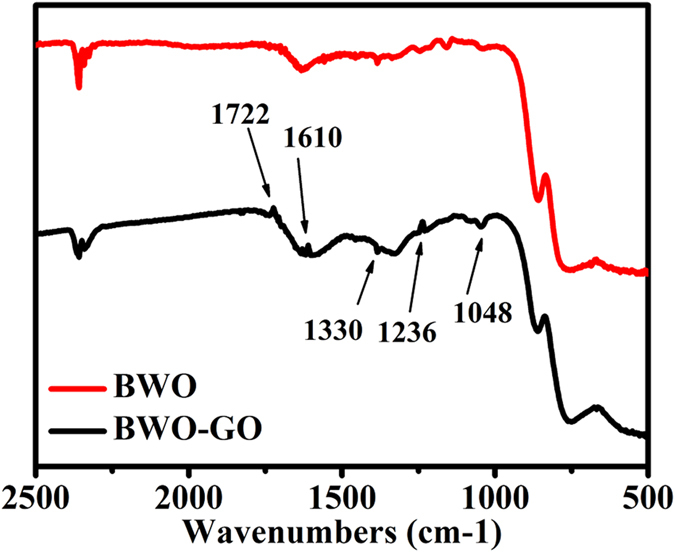
FTIR spectra of BWO and BWO-GO.

As the loaded percentage of GO very low, the GO vibration signals are very low. The FTIR spectrum in Fig. 5 displays vibration modes which corresponds to O=C-O (1330 cm^−1^), C-O-C (1236 cm^−1^), sp^2^ hybridized C=C (1610 cm^−1^), ketonic species C=O (1820 cm^−1^), carboxyl COOH (1722 cm^−1^) and hydroxyl (water) (1048 cm^−1^). The spectrum indicates the prepared graphite oxide consists of both hydrophobic (C=C) and hydrophilic (C-C, C-OH, COOH) species which makes GO amphiphilic^43^,^44^.

**Figure 6:**
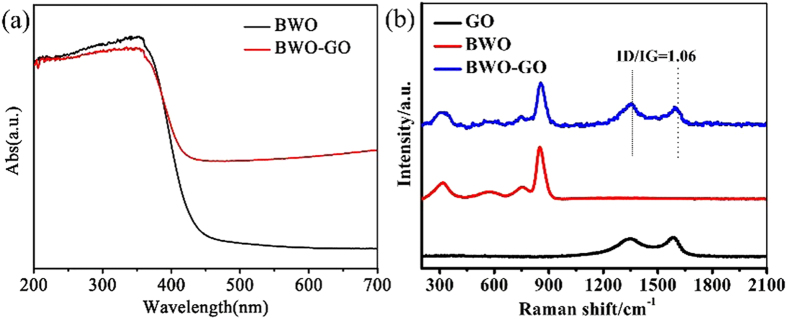
(**a**) UV-Vis absorption spectra of BWO-GO and BWO nanostructures; (**b**) Raman spectra of BWO-GO, BWO and GO nanostructures.

Optical absorbance spectrum of a semiconductor is affected by its electronic structure feature and then determines the photocatalytic activity. As is shown in Fig. 6(a), the absorption shoulder of precipitation of the precursor is found at ca. 440 nm. And a blue-shift of the optical absorption edge is observed from the UV-Vis transmission spectra, while the GO is loaded. The UV-vis spectra of the synthesis products show important features: the high absorption of the GO-loaded products in the visible range of the optical spectrum, in comparison to the previously observed GO-free nanostructured BWO.

To investigate the structural properties of the BWO-GO composites and electronic coupling between GO and BWO, we have employed Raman spectroscopy that has been established as a powerful method for the characterization of carbon nanomaterials^45^. Figure 6(b) displays the Raman spectra of the BWO-GO composites compared with the bare GO suspension and BWO at 532 nm excitations. The pristine GO exhibits the graphitic Raman G band at 1580 cm^−1^ arising from the bond stretching of sp2 carbon atoms and the defect activated D band at 1350 cm^−1^. The Raman spectra of BWO-GO shows that the two bands have similar intensity (ID/IG = 1.06) this implies a high quantity of structural defects due to the oxidation of graphite^46^. The pristine BWO exhibits two absorption peaks at 850 cm^−1^ and 325 cm^−1^. Above absorption peaks all exist in the spectra of BWO-GO composites. The clear identification of the BWO-GO heterojunction is allowed by the Raman spectroscopy.

### Photocatalytic activity test

**Figure 7:**
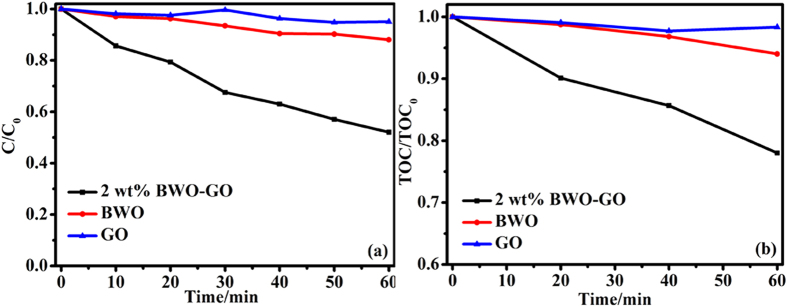
(**a**) TC removal; (**b**) TC mineralization, by 0.2 g 2 wt% BWO-GO, 60 min, under visible light irradiation.

The photocatalytic activities are tested by TC removal and TC mineralization. Figure 7 displays TC removal and mineralization by 2 wt% BWO-GO in 60 min. As is shown in Fig. 7(a), the obviously improved TC removal comes from the synergistic effect of BWO and GO. Uniformly, the obviously improved TC mineralization also comes from the synergistic effect of BWO and GO (Fig. 7b).

### Response surface methodology model analysis

The CCD design combined with the experimental and predicted data of TC photocatalytic degradation under visible light irradiation using BWO-GO nanocomposites are listed in Table 1. A second-order polynomial expression shown in Eq. (1) consisting of 10 coefficients was attained from the analysis of variance (ANOVA) at 95% confidence level (p < 0.05).


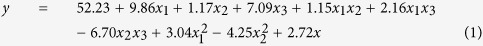


where y refers to removal efficiency expressed in % and x_1_, x_2_, and x_3_ represent the uncoded values of photocatalyst concentration, percentages of GO and reaction time, respectively.

The sum of squares, mean squares, estimated coefficient, standard error, and the corresponding F-value and p-values are also tested using ANOVA, and the results are summarized in Table 2. In statistics, a model with a large F-value (F_model_ = 23.46, much greater than unity) and a small p-value (<0.05) is considered to be significant. Furthermore, the fit of the model is verified by the coefficient of determination R^2^. In this study, the value of the determination coefficient (R^2^ = 0.9679) shown in Table 2 indicated that 96.79% of the variability in the response could be explained by the model. Also, the adjusted determination coefficient R^2^ = 0.9266 is also high indicating that the obtained model is significant.

It is observed in the ANOVA test that for the first-order main effects, x_1_ and x_3_ are more highly significant than x_2_. In contrast, for the second-order main effects, x_2_^2^ is more highly significant than x_1_^2^ and x_3_^2^. Moreover, in terms of interactive effects, x_2_ x_3_ is more significant than x_1_ x_2_ and x_1_ x_3_.

**Figure 8:**
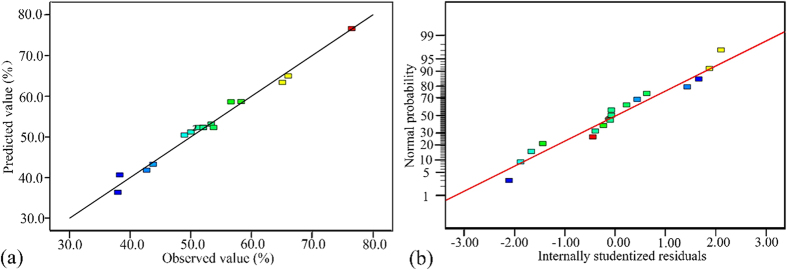
(**a**) The experimental TC degradation (%) plotted against the predicted values derived from the RSM model; (**b**) The internally studentized residuals versus normal % probability distribution.

The ANOVA reveals that Eq. (1) (R^2^ = 0.9679) suitably explains the actual relationship between the response and the variables, which can be seen in Fig. 8(a) by comparing the experimentally measured values against the predicted responses for the TC removal efficiency. Moreover, the normal probability plot of the residuals is illustrated in Fig. 8(b), and it shows that there is almost no violation of the assumptions: errors are normally distributed and independent, while the error variance is homogeneous.

**Figure 9:**
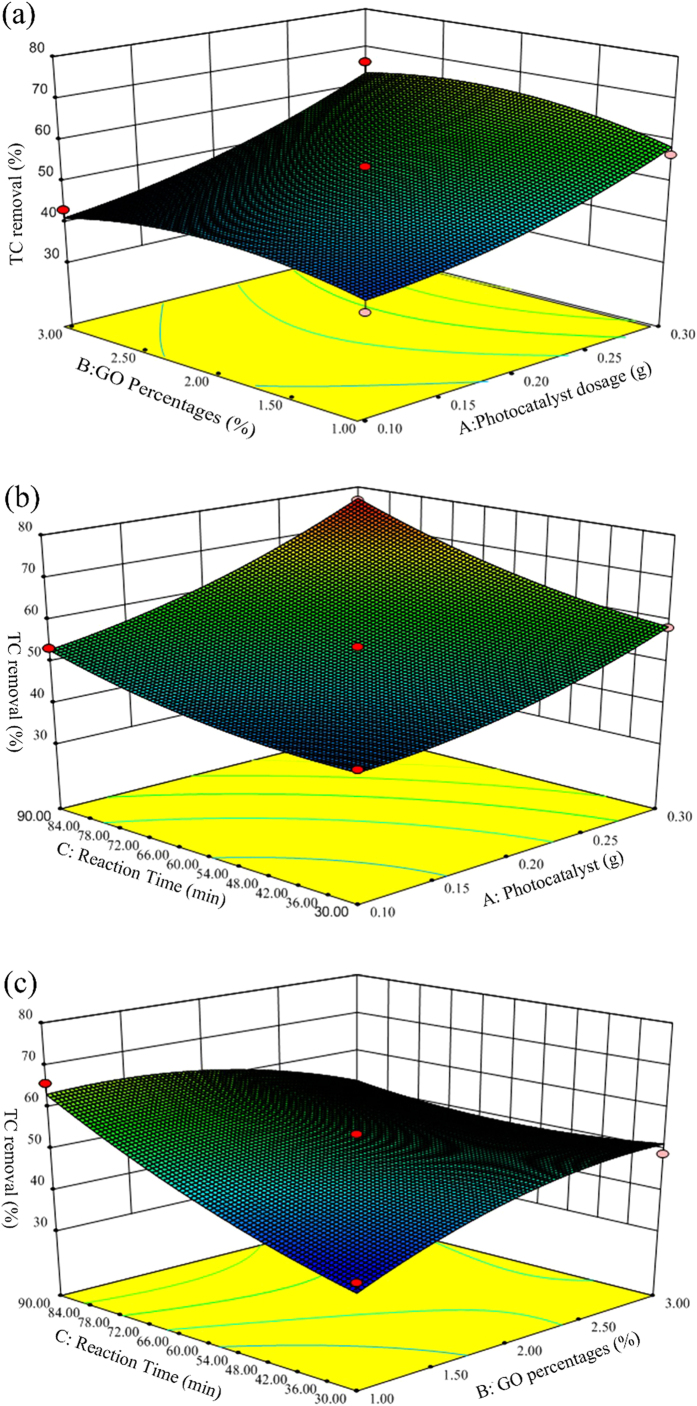
(**a**) Effect of catalyst dosage and GO percentages on TC removal; (**b**) Effect of catalyst dosage and reaction time on TC removal; (**c**) The effect of GO percentages and reaction time on TC removal.

After performing all the experiments which are suggested by CCD, the response surface analysis is carried out, in order to investigate of effects of the variables and find optimal conditions for *E. coli* disinfection. Response surface analysis helps in identification of the type of interactions between the selected variables. In Fig. 9, response surface plots of TC concentration are displayed for the three pairs of the factors. According to the Fig. 8(a) and (b), the TC removal efficiency steadily improved with the increased photocatalyst dosage of the photocatalyst dosage from 0.1 to 0.3 g. The main reason is that the number of active sites in the solution increased with catalyst dosage. Furthermore, as it can be concluded from these figures, TC removal is extremely more sensitive to changes in catalyst dosage compared with other parameters. This is in agreement with the P-values obtained for each parameter from ANOVA.

According to the Fig. 9(b) and (c), the TC removal efficiency improved with the increased reaction time of the reaction time from 30 to 90 min. Furthermore, according to P-values obtained from ANOVA, TC removal is slightly sensitive to changes in reaction time from 30 to 90 min.

According to the Fig. 9(b) and (c), the TC removal doesn’t have linear relationship with GO percentages, and the optimized GO percentage is of 1.49 wt%. The proper explanation can be that 1.49 wt% is the best percentage for photoinduced hole-electron separation between GO and BWO.

The optimal values of the selected variables are obtained using numerical optimization method provided by the Design-Expert 9.0.3 software. The goal of the optimization is to minimize the final concentration of TC in the solution. The point at which the final concentration of TC is predicted to be in its lowest value is found to be 0.3 g, 1.49 wt%, and 90 min for photocatalyst dosage, GO percentage and reaction time, respectively. Based on the suggested model (Equation (1)), final TC removal under the optimal condition should be 80.22%. To confirm the accuracy of the optimization, duplicate verification experiments are carried out. The average final TC removal in these experiments is found to be 78.43%, which is reasonably close to the predicted value. Therefore, the optimum point determined by RSM is successfully verified and suggests that RSM can be a powerful tool for optimizing photocatalytic purification processes.

### Mechanism of photocatalysis

**Figure 10:**
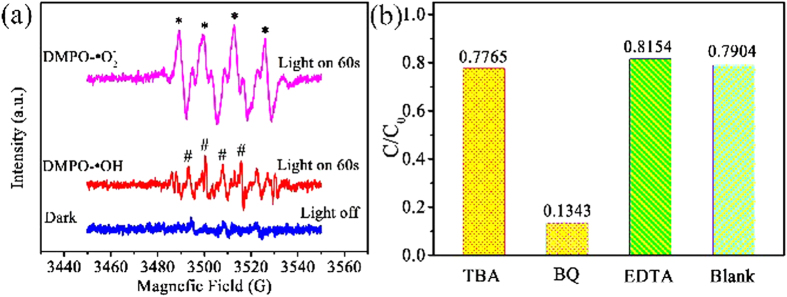
(**a**) ESR signals of the DMPO-·O_2_^−^ and DMPO-·OH. (**b**) Effects of a series of scavengers on the degradation efficiency of TC by optimized sample.

For the better application of BWO-GO photocatalyst in pollutants photocatalytic degradation, it is imperative to understand the mechanism of photocatalysis of TC under visible light irradiation. To explore the mechanism of photocatalytic degradation of TC by BWO-GO heterostructure photocatalysts, ESR spectrum and radical scavenger experiments are employed to ascertain the active species. ESR spectrums are used for the detection of the production of OH· and ·O_2_^−^. Benzoquinone (BQ) as a scavenger for ·O_2_^−^, EDTA for h^+^ and tertiary butanol (TBA) for OH· are employed to observe the direct influence on the degradation rates of each radicals. The addition of quenchers to the TC solution are all prior to the addition of photocatalysts. As a consequence of capture, photocatalytic degradation of TC will be influenced and photocatalytic efficiency is changed. The effects of a series of scavengers on the photodegradation efficiency are shown in Fig. 10. As is shown in Fig. 10(a), the degradation efficiency of TC decreases rapidly from 79.04% to 13.43% after the addition of BQ, indicating that ·O_2_^−^ is the main active species in the photodegradation process. While TBA is added, the photodegradation efficiency of TC decrease from 79.04% to 77.65%. It is indicated that ·OH is not main active species. However, while the EDTA is adding, the degradation efficiency increases a little from 79.04% to 81.54%. It may be due to the quenching of h^+^, which promotes the separation of photoinduced holes and electrons. Then the degradation efficiency is improved. The ESR spectra also reveals the proof of these phenomenon, as is shown in Fig. 10(b). In these ESR detection, 10 mg samples and 50 μL DMPO are dissolved in 0.5 mL deionized water and stirred for 10 min, which is used in the detection of hydroxyl radicals (DMPO-·OH). 10 mg samples and 50 μL DMPO are dissolved in 0.5 mL CH_3_OH and stirred for 10 min, which is used in the detection of superoxide radicals (DMPO-·O_2_^−^). Before the irradiation, there are no characteristic peak of ·OH and ·O_2_^−^. After the irradiation (60 s), the characteristic peak intensity of ·O_2_^−^ is much larger than that of OH·. It is also accord with the consequence of radical scavenger experiments, that the ·O_2_^−^ is the main active species.

**Figure 11:**
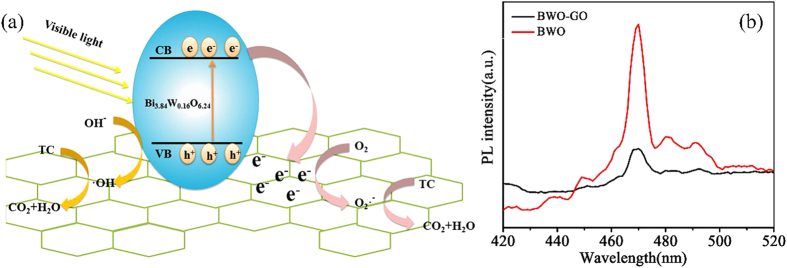
(**a**) Schematic diagram of electron-hole pairs’ separation between BWO and GO; (**b**) Room-temperature fluorescence spectra of GO and BWO-GO.

Combining the ESR spectrum and radical scavenger experiments, a possible electron transfer behavior is proposed (Fig.11(a)). According to our previous work, the CB and VB of BWO is +0.36 and +3.26 eV, separately^47^. In the photocatalytic process, electron-hole pairs are formed when BWO is irradiated by visible light, and electrons at the VB are excited to the CB, inducing the separation with holes in the VB. The existence of GO in the composite promoted the photocatalytic activity and stability. First, the high surface area of GO provides more active adsorption sites and photocatalytic reaction sites, which has the benefit of improving the photocatalytic activity. Second, GO acts as an effective acceptor of the photoexcited electrons, making ·O_2_^−^ radicals produced by the reduction of O_2_ molecules adsorbed on catalyst surface^48^.

In order to prove above-proposed mechanism, electron-hole pairs’ recombination for BWO and BWO-GO is detected by photoluminescence (PL) technique. PL is an effective approach for obtaining information about the migration and separation efficiency of the photo-generated charge carriers. The room temperature PL emission spectra of BWO and BWO-GO nanocomposites with the excitation wavelength of 320 nm are compared and illustrated in Fig. 11(b). The photoluminescence spectra are recorded over the wavelength range 420–520 nm; both samples exhibited the usual band-edge emission with a peak at 470 nm, the strongest emitting peaks around 470 nm can be attributed to the intrinsic luminescence of BWO. The peaks centered at 449, 481 and 492 nm can be attributed to the intrinsic transition of Bi^3+^, defects of metal atoms and oxygen vacancies during the crystal growing process^49^,^50^, which is become defect centers and thus affects the optical properties of BWO. It can be seen from the photoluminescence spectra that there is a significant decrease in intensity in the PL spectrum of the BWO-GO composite photocatalyst. The weaker PL intensity, the bigger possibility of photoexcited charge carrier separation^51^. Consequently, a lower PL emission intensity of the BWO-GO nano-octahedron suggests that GO can easily capture the photoexcited electrons from BWO-GO and promote the interfacial charge transfer, resulting in a lower recombination rate of the e^−^-h^+^ pairs.

## Conclusion

The BWO-GO photocatalysts have been successfully synthesized by a facile inorganic salt-assisted hydrothermal method. The octahedral BWO nanoparticles have been successfully anchored on the GO sheet. The BWO-GO heterojunction is in favor of photocatalytic activity enhancement and the main active species in TC photodegradation is ·O_2_^−^.

The optimization and the modeling of photocatalytic degradation of TC are evaluated by using a central composite design. The second order polynomials are useful in determining the optimum parameters for TC removal. The obtained optimum condition for TC removal is to achieve 80.22% with 0.3 g photocatalyst dosage, 1.49% GO loaded percentage and 90 min reaction time. This study will provide new insights for application of GO sensitized BWO photocatalytic nanocomposites for micropollutants removal from drinking water.

## Materials and Methods

### Synthesis of BWO-GO

GO was synthesized by the modified Hummers’ method and the detailed procedures could be found elsewhere^52^. In addition, a mixture of Bi(NO_3_)_3_·5H_2_O (1.0 mmol), GO and Na_2_WO_4_·2H_2_O (0.5 mmol) was dissolved in 50 mL of deionized water, followed by the addition of 2 ml of ethylenediamine (En, 99%). After being stirred for 30 min, the mixture was transferred into a 250 mL roundbottom flask in a microwave system (XH-300UL, Beijing Xiang Hu Technology Development Co. Ltd) equipped with *in-situ* magnetic stirring. After treating the mixture at 100 °C for 5 min under microwave radiation, the final products were collected using centrifugation, washed several times with deionized water and ethanol, and dried in air at 60 °C for 12 h.

### Characterization

The crystal structures of the samples are determined with the X-ray diffraction (XRD) method using Cu Kα radiation (λ = 1.54178 Å). Scanning electron microscopy (SEM) images are collected on an S-4800 field emission scanning electron microscope (Hitachi, Japan). Transmission electron microscopy (TEM), high resolution transmission electron microscopy (HRTEM) and scanning transmission electron microscopy (STEM) images are collected on an F20STWIN electron microscope (Tecnai G2, FEI Co.), using a 200 kV accelerating voltage. UV-vis diffused reflectance spectra of the samples are obtained with a UV-vis spectrophotometer (UV2550, Shimadzu, Japan); BaSO_4_ is used as the reflectance standard. The photoluminescence properties of the obtained samples are measured on a Perkin-Elmer LS 55 luminescence spectrometer, at room temperature. ESR analysis is conducted with a Bruker EPR A300-10/12 spectrometer.

### Photocatalytic degradation of TC

The photodegradation reaction for TC is carried out under simulated sunlight irradiation using a 150 W Xe lamp with a cut off filter (λ ≥ 400 nm) in a photochemical reactor under visible light. The initial TC concentration is 10 mg/L. 0.10 g of the photocatalyst is put into 100 mL of a TC solution. Before the photodegradation experiment is initiated, the suspension is magnetically stirred in the dark for 30 min to reach the absorption equilibrium. The sampling analysis is conducted in 10 min intervals. The photocatalytic degradation ratio (DR) is calculated using the following formula:


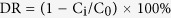


where C_0_ is the initial absorbance of TC at the absorption equilibrium, while C_i_ is the absorbance after the sampling analysis. The absorbance of TC is measured using a UV-vis spectrophotometer with the maximum absorption wavelength at 357 nm.

### Central composite design (CCD)

The central composite design (CCD) is applied to investigate the effects of the three independent variables on the response functions. The independent variables are photocatalyst concentration (X_1_), percentages of GO (X_2_) and reaction time (X_3_). The levels of the three major factors identified are summarized in Table 3. The notations (−1) and (+1) refer to the low level and the high level of the two-level-factorial design, respectively. In developing the regression equation developed by Box-Hunter, the test factors are coded according to the following equation:


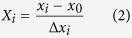


where X_i_ is the dimensionless value of an independent variable, x_i_ represents the real value of the independent variable, x_0_ is the real value of the independent variable at the center point, and Δx_i_ is the step change^53^.


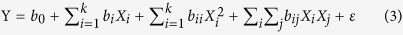


where Y is the predicted response, b_0_ and the offset term, b_i_ the linear effect, b_ij_ the squared effect and b_ii_ is the interaction effect.

## Additional Information

**How to cite this article**: Song, C. *et al*. Fabrication, Characterization and Response Surface Method (RSM) Optimization for Tetracycline Photodegration by Bi_3.84_W_0.16_O_6.24_- graphene oxide (BWO-GO). *Sci. Rep*. **6**, 37466; doi: 10.1038/srep37466 (2016).

**Publisher’s note:** Springer Nature remains neutral with regard to jurisdictional claims in published maps and institutional affiliations.

## Figures and Tables

**Table 1:** CCD design matrix for three variables with observed values for TC removal.

Lable	catalyst concentration (X_1_)	percentages of GO (X_2_)	reaction time (X_3_)	C/C_0_
1	0	0	0	52.06
2	−1	1	0	43.12
3	1	0	−1	58.30
4	0	−1	1	65.85
5	1	−1	0	56.62
6	0	−1	−1	38.00
7	−1	0	−1	43.80
8	0	0	0	52.06
9	0	1	1	50.02
10	0	0	0	52.0
11	0	0	0	53.76
12	0	1	−1	48.96
13	−1	−1	0	38.3
14	0	0	0	51.39
15	1	1	0	66.05
16	−1	0	1	53.37
17	1	0	1	75.5

**Table 2:** Results of variance analysis.

Source of variations	d.f.	Mean square	Sum of squares	F-value	p-Value
Model	9	170.33	1532.94	23.46	0.0002
x_1_	1	777.76	777.76	107.10	＜0.0001
x_2_	1	11	11	1.51	0.2582
x_3_	1	401.58	401.58	55.30	0.0001
x_1_ x_2_	1	5.31	5.31	0.73	0.4207
x_1_ x_3_	1	18.62	18.62	2.56	0.1534
x_2_ x_3_	1	179.43	179.43	24.71	0.0016
x_1_^2^	1	38.83	38.83	5.34	0.0540
x_2_^2^	1	75.99	75.99	10.46	0.0144
x_3_^2^	1	31.19	31.19	4.30	0.769
Residual	7	7.26	50.83		
Lack of fit	3	15.83	47.50	24.23	0.0079
Pure error	4	0.83	3.34		

R^2^ = 0.9679, R_adj_^2^ = 0.9266.

**Table 3:** Level and code of experimental variables.

Symbol	Variables	Coded levels		
		−1	0	+1
X_1_	photocatalyst concentration/g	0.1	0.2	0.3
X_2_	percentages of GO/%	1	2	3
X_3_	reaction time/min	30	60	90
